# Automatic metabolic breast cancer staging using [¹⁸F]FDG PET/CT: comparison with nuclear medicine physician-based and clinical staging

**DOI:** 10.1007/s00259-026-08005-y

**Published:** 2026-06-19

**Authors:** Cláudia Santos Constantino, Carla Oliveira, Francisco P. M. Oliveira, Inês Moreira, Andrea DeCensi, Susana Vinga, Durval C. Costa

**Affiliations:** 1 Champalimaud Clinical Centre, Champalimaud Foundation, Av. Brasília, Lisbon, 1400-038 Portugal; 2 Instituto Superior Técnico, INESC-ID, Universidade de Lisboa, Lisbon, Portugal

**Keywords:** Breast cancer staging, [¹⁸F]FDG PET/CT, Computer-assisted image analysis, Artificial intelligence

## Abstract

**Purpose:**

This study aimed to evaluate a deep-learning (DL)-based framework to automatically perform breast cancer (BC) metabolic staging on [¹⁸F]FDG PET/CT, and to assess agreement among DL-based, nuclear medicine (NM) physician-based, and clinical staging.

**Methods:**

A total of 403 histologically confirmed BC patients who underwent whole-body staging [¹⁸F]FDG PET/CT were retrospectively included. All [^18^F]FDG avid lesions suspected of malignancy were segmented by an NM physician and classified into four key tumor regions: primary tumor (pT), regional axillary lymph nodes (ALN), extra-axillary locoregional nodes (extra-ALN), and distant metastases (dM). Data were split into training (*n* = 303) and testing (*n* = 100) sets using a stratified approach. Class-specific DL segmentation networks were developed. A post-processing pipeline was implemented to derive metabolic TNM staging from the DL-based segmentation. Using NM-based and clinical staging segmentation as reference, accuracy, sensitivity, and specificity were computed for T, N, and M. Segmentation performance was evaluated using the Dice coefficient (DC) and lesion detection (LD) metrics. NM-based metabolic staging was also compared with clinical staging.

**Results:**

DL-based staging showed concordance with NM-based/clinical staging of 75/62%, 87/74%, and 83/82% for T, N, and M, respectively. For N3 (presence of extra-ALN) and M1 (presence of dM), where [¹⁸F]FDG PET/CT is particularly relevant, sensitivity/specificity of DL-based (NM-based reference) were 0.86/0.96, and 0.97/0.78, respectively. Segmentation performance was good to excellent for pT, ALN, and dM (median DC ≥ 0.83 and LD ≥ 0.78), and moderate for extra-ALN (median DC = 0.63 and LD ≥ 0.71). NM-based metabolic staging agreed with clinical staging in 63%, 80%, and 99% of cases for T, N, and M, respectively.

**Conclusion:**

Although expert supervision remains essential, the developed DL-based framework demonstrates potential as a supportive tool for metabolic staging in BC patients, facilitating a workflow-efficient [¹⁸F]FDG PET/CT-based staging assessment.

**Supplementary Information:**

The online version contains supplementary material available at https://doi.org/10.1007/s00259-026-08005-y.

## Introduction

Breast cancer (BC) is the most frequently diagnosed cancer in women and the second most common overall [1]. Initial treatment options may include surgery, radiotherapy, chemotherapy, targeted therapy and endocrine therapy. The decision on the optimal treatment to be pursued for a given patient depends on the tumor’s biological characteristics and the precise staging of the disease [2].

Accurate BC staging requires comprehensive assessment of disease extension and is defined from stage I to stage IV. Staging is based on the characteristics of the primary tumor (T, from T1 to T4), regional lymph node involvement (N, from N0 to N3), and the presence of distant metastases (M0 or M1), collectively referred to as the TNM classification. In clinical practice, staging follows the 8th edition of the American Joint Committee on Cancer (AJCC) staging system [3].

Whole-body [¹⁸F]FDG PET/CT is increasingly used worldwide for BC staging, particularly from TNM stages IIB to IV, and can be more helpful in more aggressive biologies such as triple-negative or HER2-positive (HER2+) BC, as it aids in detecting regional nodal involvement and distant metastases [4, 5]. BC most commonly disseminates to the ipsilateral axillary lymph nodes (level I and II of the axilla), but may also involve ipsilateral extra-axillary loco-regional nodes, such as the ipsilateral infraclavicular (level III of the axilla), supraclavicular, and/or internal mammary lymph nodes. The presence of these extra-axillary loco-regional nodes may result in upstaging of the N category according to TNM classification and, consequently, to modifications in treatment planning. When BC spreads to contralateral axillary, supraclavicular, and/or internal mammary nodes, or to ipsilateral or contralateral cervical nodes, or to any distant organ, the patient is classified as having distant metastasis (M1), which may result in upstaging and corresponding modifications to the treatment plan. [¹⁸F]FDG PET/CT has high accuracy in detecting involvement of these nodal and distant sites and has been shown to surpass other imaging modalities [6].

Deep learning (DL) models have been employed to aid in BC, using different imaging modalities, to improve different tasks, such as lesion classification and segmentation, image reconstruction and generation, cancer risk prediction, and prediction and assessment of therapy response [7, 8]. In [¹⁸F]FDG PET/CT, DL has been used to detect disease in BC patients [9] and to segment BC lesions in longitudinal studies [10]. Plus, in the paper by Li et al. [11], DL is used to assist clinicians in diagnosing axillary lymph node metastasis. The sensitivity of the physicians to diagnose axillary lymph node metastasis increased 7.8% with the aid of their proposed network [11].

As staging whole-body [¹⁸F]FDG PET/CT imaging is being increasingly used, this study evaluates the feasibility of using a DL-based aiding tool to automatically and accurately identify, segment, and classify BC disease involvement using [¹⁸F]FDG PET/CT, facilitating accurate metabolic TNM staging. In addition to the comparison between the nuclear medicine physician-based (NM-based) identification and classification and the DL-based approach, both metabolic staging methods were further compared with clinical TNM staging.

## Materials and methods

### Patient cohort

This retrospective and dual-scanner study included 403 histologically confirmed BC patients (female, mean age: 56 years; age range: 23–86 years) with available staging whole-body [^18^F]FDG PET/CT scans acquired at Champalimaud Clinical Centre, Champalimaud Foundation, Portugal. All patients were scanned after intravenous injection of 238 ± 36 MBq of [^18^F]FDG (on average 3.5 MBq/kg), either on Philips Gemini TF 16 (*n* = 177) or on Philips Digital Vereos (*n* = 226). Image acquisition and reconstruction followed EARL1 standards for ^18^F and all images were acquired with isotropic voxels (4 × 4 × 4 mm^3^). Patient selection was done ensuring representativity of the different T, N, and M staging categories, according to the clinical TNM staging. Given the relatively low incidence of N3 disease (10–12% in locally advanced breast cancer [12]), and M1 disease (approximately 5% [13]), at diagnosis, the cohort does not fully reflect real-world distribution. However, this sample strategy ensured adequate representation of these less common categories to provide sufficiently sized training and testing sets for the DL-based aiding tool. In total, 62 patients had N3 involvement and 105 had M1 disease.

The study was approved by the Ethics Committee of Champalimaud Foundation, and the need for written informed consent was waived in the terms defined by the ethics approval.

Clinical and pathological data were retrospectively retrieved from the institutional electronic medical records: patient age at time of diagnosis; menopausal state; BC histological subtype; positivity/negativity of estrogene and progesterone receptors; human epidermal growth factor receptor 2 (HER2) immunohistochemistry (IHC) overexpression or amplification; Ki67 index; tumor grade (1–2 vs. 3); clinical tumor stage (cT, from cT1 to cT4); clinical nodal status (cN, from cN0 to cN3); and, clinical metastatic status (cM0 vs. cM1). Clinical staging (cTNM) was assigned by the institutional multidisciplinary oncology team following the AJCC 8th edition staging guidelines, incorporating physical examination findings, imaging evaluation, and available biopsy results.

Across the entire dataset, ductal of no special type BC (ductal NST BC) accounted for 80% of cases, with the remainder comprising lobular, apocrine, mucinous, and micropapillary subtypes, as well as mixed histologies. Overall, 20% of patients had triple-negative breast cancer, 68% had positive hormone receptors and 29% had HER2 overexpression or amplification. Ki-67 values ranged from 10 to 95%.

For the development of the DL-based tool, a stratified random train–test split of approximately 75%/25% was performed according to class presence within each patient scan, resulting in 303 [^18^F]FDG PET/CT scans for training. The remaining 100 scans were used exclusively for independent testing of the DL-based tool. For the train-test split, the entire dataset was initially stratified by staging category, followed by a random selection within each class to prevent selection bias. This approach ensured that the distribution of tumor characteristics remained well-balanced between the training and testing sets. Detailed patient and tumor characteristics separated by train and test set are summarized in Table 1.

**Table 1:** Patients and tumors’ characteristics

	Train set (*n* = 303)	Test set (*n* = 100)
Age (mean ± SD)	55 ± 12	57 ± 12
Pre-menopausal status (%)	40	36
BC histological type (%) Ductal NST Lobular Aprocrine, mucinous or micropapillary Mixed Histologies	81 14 2 3	75 8 10 7
Bilateral BC (%)	4	3
Positive Estrogen receptors (%)	69	62
Positive Progesterone Receptors (%)	60	54
HER2 overexpression/amplification (%)	27	36
Triple-negative BC (%)	21	21
Ki67 (mean ± SD)	46 ± 24	48 ± 23
Grade (%) 1–2 3 Unknown	54 42 4	63 35 2
Clinical T stage (%) cT1 cT2 cT3/cT4	13 47 40	20 47 33
Clinical N stage (%) cNx (cannot be assessed) cN0 cN1 cN2 cN3	2 22 43 18 15	1 27 46 9 17
Clinical M stage (%) cM0 cM1	74 26	74 26
Acquisition scanner Philips Digital Vereos (%)	45	89

SD: standard deviation; NST: no special type

### NM physician-based imaging annotations

An NM physician with more than 10 years of experience identified all [^18^F]FDG avid lesions suspected of malignancy and classified them according to their anatomical location. This identification involved defining a three-dimensional region-of-interest (ROI) for each lesion, including the target lesion and surrounding background. Non-malignant high-uptake regions were specifically excluded from the defined ROI. This ensured that subsequent segmentation was restricted to specifically defined suspicious lesions, thereby minimizing potential segmentation errors a priori. After annotation, each identified lesion was independently and semi-automatically segmented using a robust adaptive Bayesian classifier [14]. The segmentation was reviewed by an experienced observer and adjusted when necessary. This final segmentation was used as ground truth for DL network training and testing.

The physician was blinded to all patient clinical information (including pathological results) and had access only to the imaging findings described in the [^18^F]FDG PET/CT report, excluding the clinical history. Lesions were categorized into four clinically relevant classes reflecting key tumor regions for BC staging: (1) primary tumor (pT); (2) axillary lymph nodes (ALN; level I-II of the axilla, ipsilateral and contralateral); (3) extra-axillary locoregional lymph nodes (extra-ALN; level III of the axilla, supraclavicular and/or internal mammary chains, ipsilateral and contralateral); and (4) distant metastases (dM). Only for model optimization, ipsilateral and contralateral nodes were grouped within the same class; however, for TNM classification, contralateral lymph nodes are considered as M1.

### DL-based tool

#### Segmentation models development

DL networks were trained to identify and segment each class using a two-channel (PET and CT) 3D U-Net. In total, four separate networks were trained, yielding four models to be integrated into the DL-based tool: (1) pT; (2) ALN; (3) extra-ALN; and (4) dM. A single multi-class model was not pursued due to the significant class imbalance between the pT (303 cases), ALN (246 cases), extra-ALN (68 cases), and dM (81 cases). Preliminary experiments indicated that a multi-class approach would not achieve satisfactory performance across all categories, as the network disproportionately optimized for the more prevalent classes. The state-of-the-art nnU-Net framework was used to train the models and their respective inference. nnU-Net was proposed by Isensee et al. for medical image segmentation and automatically adapts itself to any new medical imaging dataset used [15]. Outstanding segmentation results from models trained with nnU-Net have been published with application to [^18^F]FDG PET/CT imaging [16, 17]– [18]. CT was resampled to PET isotropic spacing. For the PET data, image intensity normalization was based on z-scoring. The default nnU-Net configurations were used. The training of each network was performed with five-fold cross-validation and trained over 1000 epochs, each defined with 250 training iterations. For the optimizer, stochastic gradient descent with a large Nesterov momentum of 0.99 and a high initial learning rate of 0.01 was used for learning network weights. The loss function used is a combination of Dice loss and cross-entropy loss, which are simply averaged. Models’ training and inference were performed in a computer equipped with NVIDIA RTX A6000 graphics.

#### Staging procedure step

**Fig. 1:**
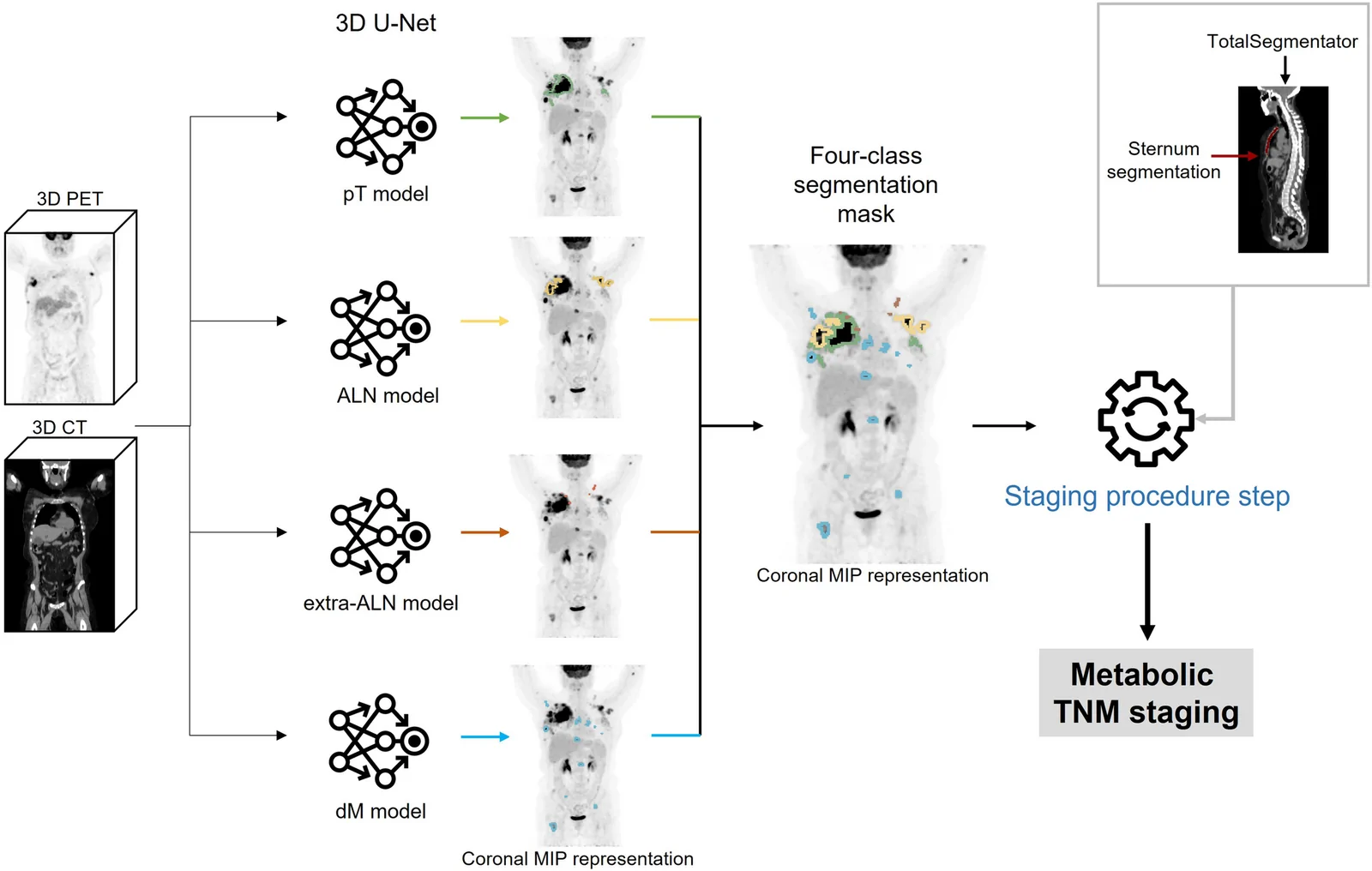
Workflow schematic of the developed DL-based tool for metabolic TNM staging based on [^18^F]FDG PET/CT scans

After [^18^F]FDG PET/CT segmentations inferred by each class-specific model, they are then combined to generate a final four-class segmentation mask. To proceed with TNM staging calculation, a staging procedure step was developed (Fig. 1). Development and optimization of this procedure were done in a subset of the training dataset. Metabolic TNM staging was calculated based on the 8th edition of the AJCC staging system. Given that the AJCC criteria are primarily designed for clinical and pathological assessment, specific adaptations were implemented where a direct correspondence between AJCC system definitions (e.g., physical examination findings) and metabolic imaging-derived information was not possible. To enable TNM staging, ipsilateral and contralateral ALN and extra-ALN are first distinguished with respect to the pT location. For this purpose, the sternum is used as an anatomical reference. The sternum is automatically segmented based on the whole-body CT scans using the published TotalSegmentator model developed by Wasserthal et al. [19]. TotalSegmentator was trained to segment anatomical structures in CT scans, including the sternum, with excellent results. After applying TotalSegmentator, the coordinates of the sternum segmentation, together with those of the pT segmentation, are used to separate ipsilateral from contralateral ALN and extra-ALN. Ipsilateral ALN and extra-ALN are considered for N staging, whereas contralateral nodes are classified as dM. Following this separation, the number of lesions per class may be quantified. These counts are subsequently used to determine the number of ipsilateral ALN relevant for N staging. Specifically, 1–3 involved ipsilateral ALN are classified as N1, and 4–9 involved ALN as N2, following Nijnatten et al. [20] and the 8th edition of the AJCC staging system for pathologic N staging. Direct correspondence from [¹⁸F]FDG PET/CT to clinical N1/N2 staging is not possible (differentiate movable and fixed or matted nodes), so this classification was used. The presence of lesions categorized as ipsilateral extra-ALN results in classification as N3. Note that there were no patients in the entire dataset with ipsilateral extra-ALN without concomitant ipsilateral ALN involvement. If lesions are categorized as dM, the M category is assigned as M1. For T staging, the largest distance among the segmented voxels of the pT segmented is calculated and used for metabolic T classification: T1 for lesions ≤ 20 mm, T2 for lesions > 20 mm and ≤ 50 mm, and T3/T4 for lesions > 50 mm. If BC is bilateral, separate T and N stages were defined for each ipsilateral side using the same criteria.

### NM-based metabolic staging

For NM-based metabolic staging, the semi-automatically generated ground truth segmentations were used. The resulting four-class segmentation mask was incorporated into the staging procedure step to derive the metabolic NM-based TNM staging.

### Statistical analysis

Statistical analysis was performed using R software (version 3.2.5, https://www.r-project.org/).

As an initial evaluation for the performance of the DL models on the test set, class-wise segmentation performance was assessed using the Dice coefficient (DC), which measures the overlap between the ground truth (NM-based segmentations) and the DL-based segmentation for each class. 0 means no overlap, and 1 means perfect overlap. Furthermore, class-wise lesion detection metrics were calculated, namely: lesion detection sensitivity (LDS) and lesion detection positive predictive value (LDPPV).

Clinical, NM-based, and DL-based TNM staging were compared pairwise based on accuracy, sensitivity, and specificity separately for T, N, and M categories. 95% confidence intervals (95% CI) were also calculated for accuracy.

## Results

### Segmentation performance of the DL-based models

**Fig. 2:**
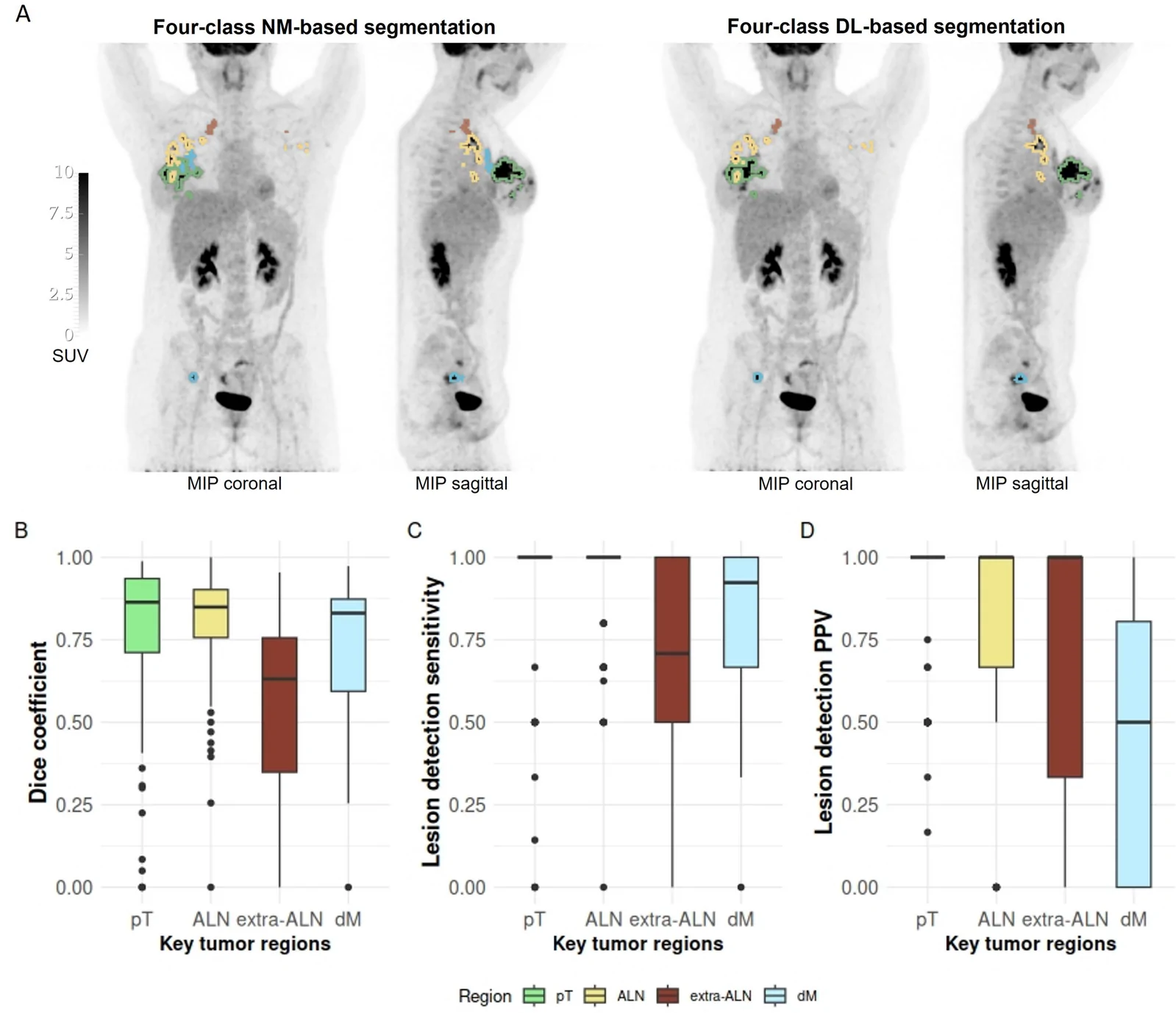
(**A**) Coronal and sagittal maximum intensity projection images (MIP) of a patient in the test set overlapped with four-class segmentations: on the left, from the nuclear medicine physician-based annotations – NM-based segmentation –, and on the right, from the four DL-based models – DL-based segmentation –. (**B**, **C**, and **D**) Box plots representing the dispersion of the Dice coefficient, lesion detection sensitivity, and lesion detection positive predictive values (PPV), respectively, in the test set for each class (primary tumor (pT), axillary lymph nodes (ALN), extra-axillary loco-regional lymph nodes (extra-ALN), and distant metastases (dM)) calculated between NM-based and DL-based segmentations

Automatic identification, segmentation, and classification performance was assessed in the test set with DC, LDS and LDPPV calculation between the four-class segmentation mask using the four DL models and the ground truth (based on the nuclear medicine physician identifications and segmentation). An example of the four-class segmentation results from a patient in the test set is shown in Fig. 2 (A). The number of lesions per patient varied by anatomical region: ranging from 1 to 7 for pT, 0 to 10 for ALN, 0 to 5 for extra-ALN, and 0 to 50 for dM.

Median and interquartile range (IQR) of DC, LDS and LDPPV were calculated for each class. The dispersion of the DC, LDS and LDPPV results per class is shown in Fig. 2 (B, C and D, respectively). Good to excellent performance was achieved for pT (median[IQR] DC, LDS and LDPPV were 0.86[0.22], 1.00[0.00], and 1.00[0.00], respectively). In 3 out of the 100 patients, the DL model failed to detect any pT lesion. These corresponded to pT lesions with maximum standardized uptake values (SUV_max_) between 1.6 and 2.5.

Good performance was also achieved for the ALN class with a median[IQR] of DC, LDS and LDPPV of 0.85[0.15], 1.00[0.00], and 1.00[0.33], respectively. The DL model failed to detect any ALN in one patient, where the ALN lesion had an SUV_max_ of only 2.1.

The performance was only moderate for extra-ALN, with a median[IQR] of DC and LDS of 0.63[0.50] and 0.71[0.5], respectively. Although the median LDPPV was 1.00, the wide IQR of 0.83 indicates performance variability across the whole test set. This may be explained by the small volumes of these lesions compared to the whole volume (minimum and maximum volume of lesions are 0.26 cm^3^ and 20 cm^3^, respectively), as well as the uptake (minimum and maximum SUV_max_ are 1.9 and 20.5, respectively). Additionally, in the entire dataset (403 patients), only 184 extra-ALN lesions were identified (148 in the training set and 36 in the test set), and overall, with a small volume (median of 1.53 cm³), which likely limits training.

In 4 out of the 20 patients in the test set with positive extra-ALN lesions according to ground truth, no lesion was identified by the DL-based model. In these, only one extra-ALN lesion was identified per patient in the ground truth, and their volume and SUV_max_ varied from 0.09 cm^3^ to 2.9 cm^3^ and from 1.9 to 2.9, respectively.

Among the 25 patients in the test set with dM identified in the ground truth, the DL model failed in identifying any lesion in only one patient (a single dM lesion with diffuse uptake in a vertebra, SUV_max_= 3.2). For that 25 patients, the median[IQR] of DC, LDS and LDPPV for dM was of 0.83[0.28], 0.92[0.33], and 0.78[0.32], respectively. However, in 16 patients out of 100, dM was identified by DL-based model, but not in the NM-based segmentation, leading to a global LDPPV of 0.5[0.82].

### Metabolic TNM staging performance

#### DL-based staging versus NM-based staging

There were three bilateral tumors in the test set; thus, the evaluation of TNM staging was performed in 103 cases. Among these three bilateral BC, the DL model correctly detected bilateral disease in two patients. In these two cases, T, N, and M bilateral staging showed 100% correspondence between NM-based staging and DL-based staging. In 10% of patients (10 out of 100 patients in the test set), the DL-based tool identified bilateral pT uptake that was not reported in the NM-based annotations. This resulted in a bilateral T stage according to the DL-based staging. Even so, only in one of these cases DL-based models detected bilateral ALN, leading to a bilateral N1 stage, whereas NM-based staging defined unilateral N1 disease. In the remaining cases, bilateral pT uptake identified by the DL-based tool did not result in a change in N classification. M staging by the DL-based tool was not affected by this.

In total, DL-based staging achieved a multi-class concordance for T, N and M of 75%, 87% and 83% compared with the NM-based staging, respectively. The three patients reported in the previous subsection, in whom the DL-based model failed to identify any pT, plus the one missed contralateral pT lesion in a patient with bilateral disease, were excluded from the T staging sensitivity and specificity calculation.

**Fig. 3:**
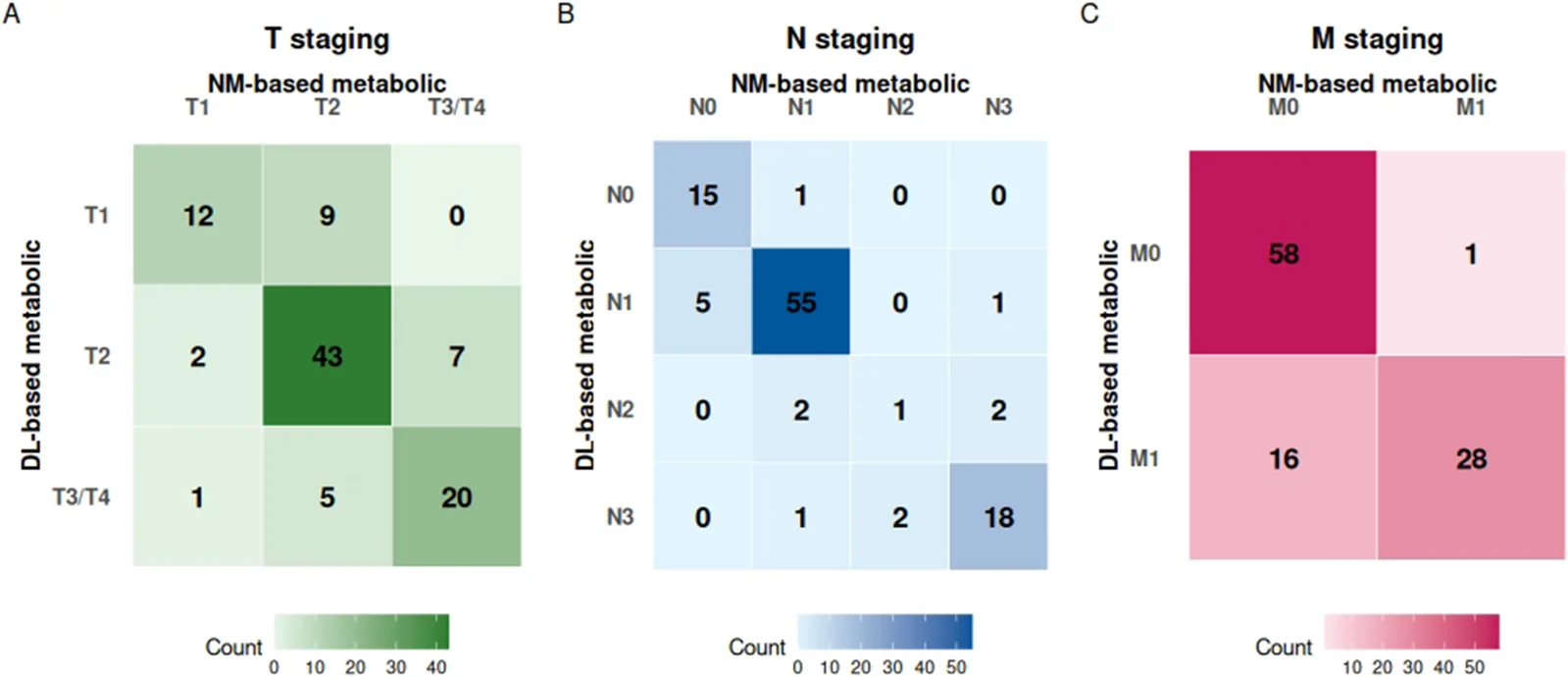
Confusion matrices between NM-based staging (reference) and DL-based staging (predicted class) for T, N, and M staging in the 103 primary tumors (subfigures A, B and C, respectively). Note that for T staging only 99 cases were used (3 cases were excluded because the DL-based failed to detect primary tumor, plus 1 missed contralateral primary tumor lesion in a patient with bilateral disease); for N and M staging all 103 cases were included

Table 2 shows the results for the sensitivity and specificity for stage classification of the DL-based staging relative to NM-based staging. In Fig. 3, the corresponding confusion matrices for T, N and M classifications are reported. Good to excellent sensitivity and specificity were found for T and N staging (≥ 0.74), except for N2 classification. Only 3 patients were N2 based on NM-based staging, but, for two of them, the DL-based tool led to upstaging to N3. Overall, three patients were upstaged from N1/N2 to N3 by the DL-based tool. The misidentification of the ipsilateral extra-ALN occurred in one case at axillary level II, where separation from level III was challenging, and in two cases involving mediastinal nodes located very close to the external mammary chain. For M staging, DL-based identified M1 patients with a sensitivity and specificity of 0.97 and 0.78, respectively. In 16 patients, the DL-based tool upstaged M0 to M1. In these, dM was identified due to uptake in the contralateral axilla (*n* = 9), and intense uptake in brown adipose tissue adjacent to the vertebrae (*n* = 4) and in thyroid (*n* = 3). These findings are plausible false positives due to the known inherent uptake of [¹⁸F]FDG in non-malignant regions with high glucose consumption, which is a known pitfall in [¹⁸F]FDG PET/CT interpretation. Nevertheless, such regions are typically easy to identify and correct with expert human review. Notably, only one patient was downstaged from M1 to M0 by the DL-based approach (the case with a single dM lesion with diffuse uptake in a vertebra, SUV_max_= 3.2).

**Table 2:** Results of multiclass accuracy for T, N, and M staging, as well as sensitivity and specificity calculated per T, N, and M subclassification, derived from the DL-based tool and compared with the NM-based staging

	Sensitivity	Specificity	Multi-class accuracy (95% CI)
T stage			
T1	0.80	0.87	0.75 (0.66; 0.84)
T2	0.75	0.81	
T3/T4	0.74	0.94	
N stage			
N0	0.75	0.99	0.87 (0.78; 0.92)
N1	0.93	0.82	
N2	0.33	0.98	
N3	0.86	0.96	
M stage			
M0	0.78	0.97	0.83 (0.75; 0.90)
M1	0.97	0.78

**Fig. 4:**
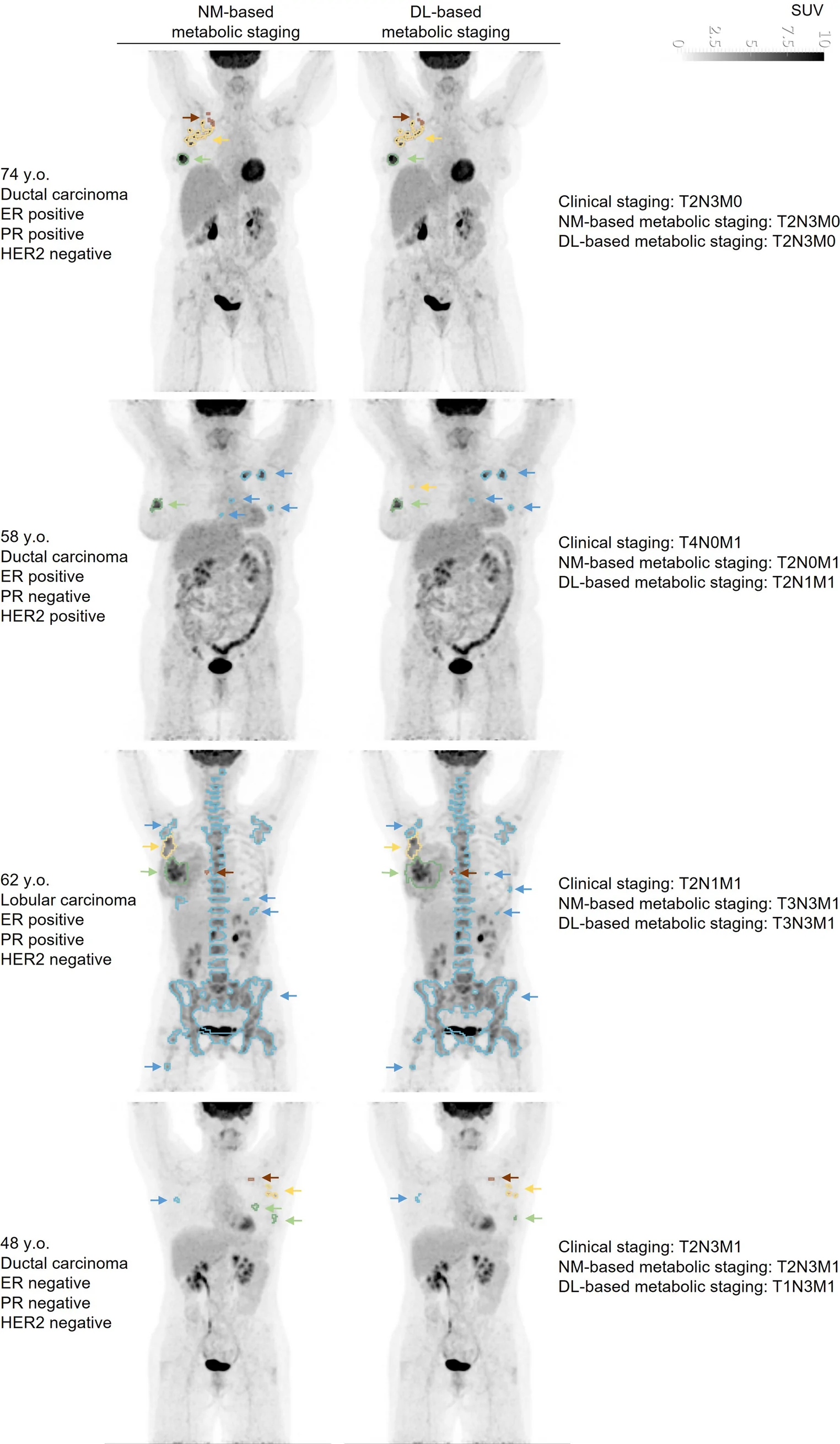
Coronal maximum intensity projection images of four patients from the test set, overlaid with the final segmentations obtained after processing by the staging framework, from which both NM-based staging and DL–based staging were derived. (ER: estrogen receptor; PR: progesterone receptor; HER2: human epidermal growth factor receptor 2)

Figure 4 shows four representative examples from the test set, including the final segmentations used for staging after processing by the staging procedure step, together with the corresponding TNM staging based on the DL-based tool and NM-based, as well as the clinical staging.

#### DL-based staging versus clinical staging

The DL-based tool was trained to replicate NM physician assessment. Nevertheless, when compared with clinical TNM staging, it also demonstrated good concordance for T, N, and M (62%, 74%, and 82%, respectively).

**Fig. 5:**
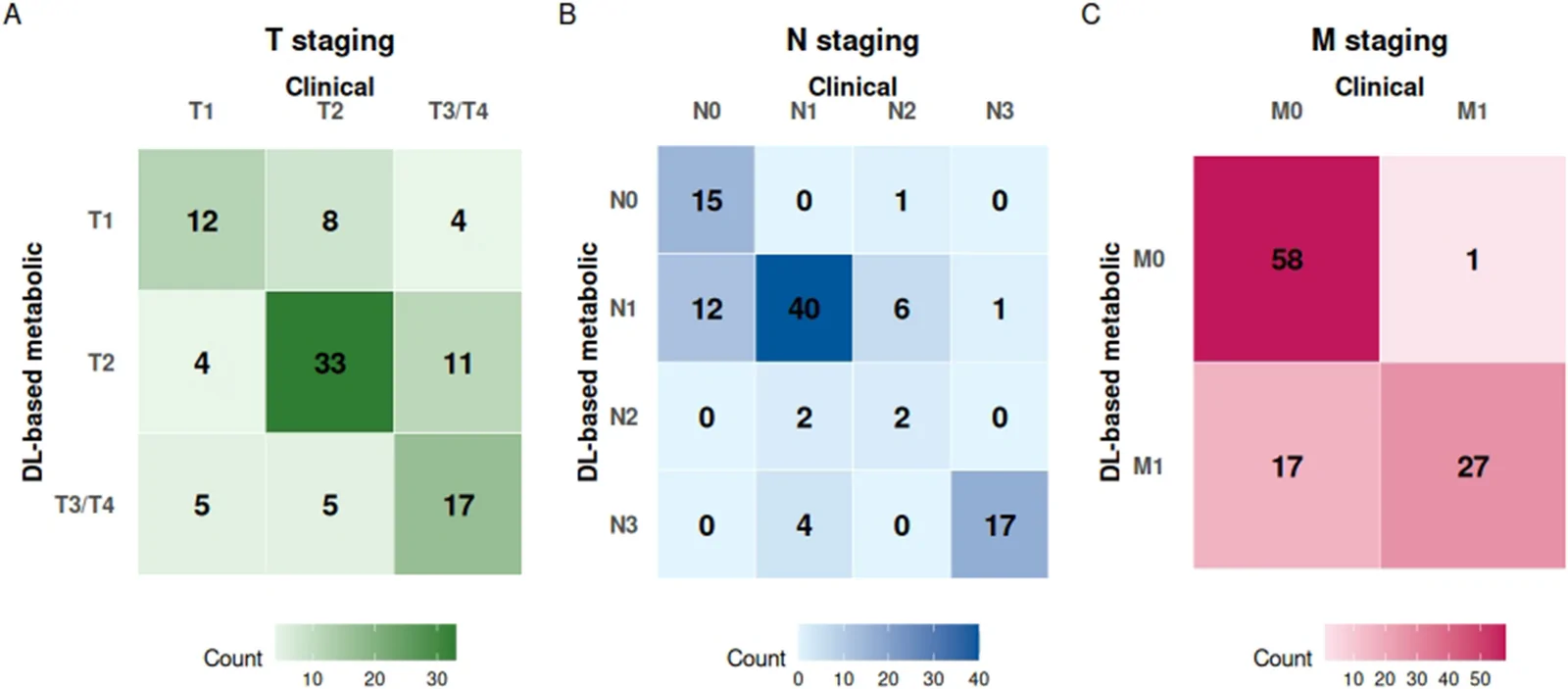
Confusion matrices between clinical staging (reference) and DL-based staging (predicted class) for T, N and M staging in the 103 primary tumors (subfigures A, B and C, respectively). Note that for T staging only 99 cases were used (a lobular carcinoma in situ excluded without TN staging and more 3 cases excluded because the DL-based did not detect primary tumor); for N staging only 101 cases were included (1 lobular carcinoma in situ was excluded because TN staging was not defined, and a Nx); for M staging all 103 cases were included

Table 3 summarizes the sensitivity and specificity of DL-based stage classification relative to clinical staging. For T staging, sensitivity and specificity—using clinical stage as reference—were identical to those observed when comparing clinical and NM physician-based staging. Figure 5 shows the corresponding confusion matrices for T, N, and M classifications. N stage classification showed similar performance to that observed between clinical and NM-based staging (good-to-excellent sensitivity and specificity across N subcategories, except for lower sensitivity in N2). We highlight N3 automatic classification by the DL-based tool, where [¹⁸F]FDG PET/CT plays a particularly relevant role, with sensitivity and specificity of 0.94 and 0.95, respectively. DL-based M stage classification demonstrated similar performance relative to both clinical and NM physician-based staging. For M1, sensitivity and specificity were 0.96 and 0.77 when compared with clinical staging, and 0.97 and 0.78 when compared with NM-based staging.

**Table 3:** Results of multiclass accuracy for T, N, and M staging, as well as sensitivity and specificity calculated per T, N, and M subclassification, derived from the DL-based tool and compared with the clinical staging

	Sensitivity	Specificity	Multi-class accuracy (95% CI)
T stage			
T1	0.57	0.85	0.62 (0.51; 0.71)
T2	0.72	0.66	
T3/T4	0.53	0.90	
N stage			
N0	0.56	0.99	0.74 (0.64; 0.82)
N1	0.87	0.63	
N2	0.22	0.99	
N3	0.94	0.95	
M stage			
M0	0.77	0.96	0.82 (0.73; 0.89)
M1	0.96	0.77	

### NM-based metabolic versus clinical staging

When comparing clinical staging with metabolic NM-based staging, concordances of 63%, 80%, and 99% for T, N, and M staging, respectively, were achieved. It should be noted that clinical T staging is essentially defined with MRI. Conversely, N and M clinical staging already incorporates [¹⁸F]FDG PET/CT reports, which is particularly relevant for cN3 and cM1 classification. Detailed results of this comparison are in the Supplementary Materials.

**Fig. 6:**
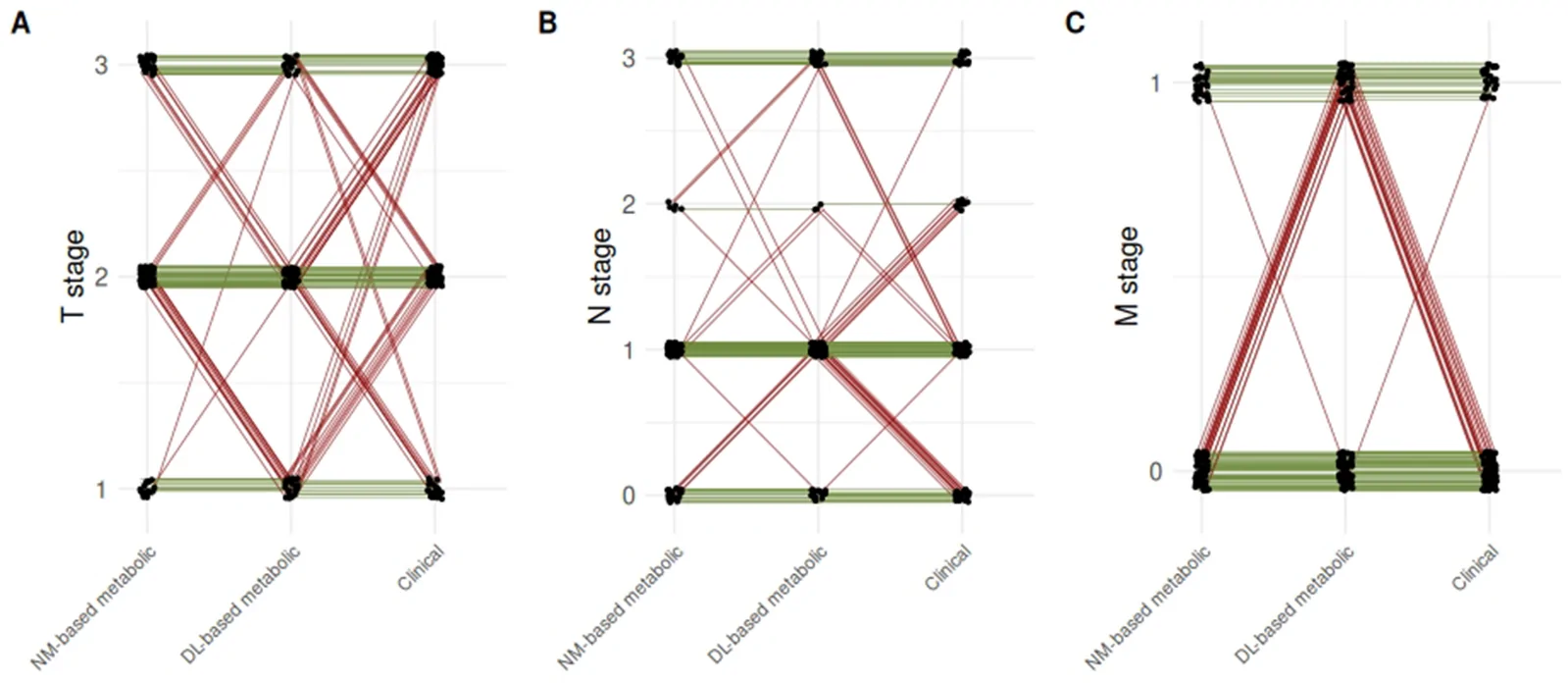
Parallel correspondence between NM-based, DL-based and clinical staging for T (left), N (middle), and M (right) staging. Please note that almost all discrepancies in T and N staging involved only a one-level difference, with cases classified as either the adjacent lower or higher stage

Figure 6 illustrates the paired correspondence between NM-based, DL-based and clinical staging for T, N, and M categories.

## Discussion

To the best of our knowledge, this is the first study to develop an automatic tool for BC staging based on [¹⁸F]FDG PET/CT that simultaneously detects and classifies the four key tumor regions paramount for staging and subsequent treatment decision-making: pT, ALN, extra-ALN, and dM. The dataset used in this study includes a higher proportion of extra-ALN and dM cases than their real-world incidence. This ensures sufficient representation of these less common categories for training and evaluation, providing greater confidence in the tool’s performance, even in these less common scenarios.

To ensure our staging framework performs comparably to an NM physician interpreting [¹⁸F]FDG PET/CT scans, we trained our models using physician-based annotations, which included both lesion identification and classification into the four defined classes, and subsequently developed our framework based on these models. When evaluating segmentation performance prior to assessing final staging, the DL-based models showed good agreement with the physician-based segmentations for pT, ALN, and dM, with a median DC ≥ 0.83. Median LDS was ≥ 0.92, and median LDPPV ≥ 0.78, considering the true positive cases. Interestingly, the only instance in which the DL model failed to detect any positive ALN, according to the physician, was also one of the three cases where no pT was detected by the corresponding DL model (a cT1cN1cM0 BC-NST, with positive hormone receptors and HER2 overexpression). In addition, the DL model detected a dM lesion in this case, whereas the physician had not identified one; this corresponded to a case with very intense uptake in brown adipose tissue. This was the only case in which the DL models failed to detect all positive classes.

The median DC for extra-ALN was moderate (0.63), although median LDS and LDPPV was 0.71 and 1.00, respectively. DL models failed to detect any extra-ALN lesions in only four patients. For those patients, the missed lesions had volumes ranging from 0.09 to 2.9 cm³ and SUV_max_ values between 1.9 and 2.9. Our median DC results, despite addressing a more complex task involving four different classes, fall between those reported by Moreau et al. [10], who reported a mean DC of 0.66 for segmenting baseline metastatic BC patients and by Leal et al. [9], who achieved a mean DC of 0.91 when automatically segmenting lesions in a smaller early-stage BC dataset.

The proposed DL-based pipeline achieved concordance rates of 75%, 87%, and 83% for T, N, and M, respectively, compared with the NM-based staging. Since [¹⁸F]FDG PET/CT is particularly important for detecting regional nodal involvement and distant metastases, it is especially relevant to highlight the concordance for N3 and M1. For these categories, the DL-based framework achieved a sensitivity and specificity of 0.86 and 0.96 for N3, and 0.97 and 0.78 for M1, respectively. Despite these high-performance metrics, a potential impact on patient care remains in specific cases without expert supervision. Three patients were upstaged from N1/N2 to N3 in challenging anatomical regions, and 16 patients were upstaged to M1—either due to non-malignant contralateral ALN uptake or intense uptake in both thyroid and brown adipose tissue. These cases could be easily corrected with expert supervision, which remains essential. For further clinical implementation, increasing the representation of these common false-positive patterns within the training set may improve the performance of the DL-based M1 staging. Additionally, one false-negative M1 case—involving one bone lesion with diffuse, low uptake identified by the NM physician— out of 25 positive cases was classified as M0 by the DL-based approach.

Similar accuracy was observed for clinical versus DL-based staging, comparable to that of clinical versus NM-based staging: 62% versus 63% for T and 74% versus 80% for N, respectively. Some level of discordance between clinical T staging and both NM-based and DL-based staging is expected and not necessarily indicative of errors, as metabolic T classification is biologically distinct from anatomical T staging. Metabolic T staging may therefore provide complementary information for clinical assessment rather than competing with conventional clinical T staging. A slightly different pattern was observed for M staging, where lower accuracy was observed for M staging when comparing DL-based with clinical staging (82%), compared with 99% for clinical versus NM-based staging. DL-based versus clinical staging accuracy for M staging is consistent with the DL-based versus NM-based comparison (accuracy of 83%). Note that the high level of agreement between clinical and NM-based staging is especially expected for N3 and M. This, given that clinical staging already incorporates [¹⁸F]FDG PET/CT information, especially due to the high accuracy of [¹⁸F]FDG PET/CT for detecting nodal involvement and distant metastasis.

For the NM physician’s lesion identification, the physician was blinded to all patient clinical information, which does not reflect real-life interpretation conditions. However, we chose this approach for two main reasons: (a) to avoid possible data leakage, and (b) to ensure that the identification of suspicious malignant lesions was based strictly on the imaging data, including findings described in the [^18^F]FDG PET/CT report. Lesion identification relied on physician expertise and their clinical judgment. We believed our option ensured a fair comparison between the NM physician’s interpretation and the DL-based approach.

This study has some limitations. In addition to its retrospective and single-center design, lesion identification and classification were performed by a single NM physician. Future validation studies should therefore evaluate inter-observer variability and incorporate multi-reader consensus to establish a more robust ground truth for model training and testing. Furthermore, for clinical TNM staging classification and comparison, histopathological confirmation was not available for all lesions; biopsies were performed for all primary tumors and for some axillary lymph nodes, but not for all suspected metastatic sites. Lastly, while our tool is based on the 8th edition of the AJCC TNM staging system, certain adaptations were necessary where clinical definitions lacked a direct correlate in [¹⁸F]FDG PET/CT imaging. This lack of direct correspondence may account for discrepancies between clinical and metabolic TNM staging (affecting both NM-based and DL-based results), particularly regarding the differentiation between N1 and N2 stages. Also, since our dataset did not include patients with positive internal mammary lymph nodes in the absence of axillary lymph node involvement, this scenario was not incorporated into the staging framework. In such cases, our framework would classify them as N3, whereas the 8th edition of the AJCC system would stage them as cN2.

Despite previous studies reporting good performance of DL-based tools for BC [¹⁸F]FDG PET/CT whole-body tumor segmentation [8–10, 21], the present study goes beyond tumor burden segmentation by integrating anatomical location to classify tumors and derive a metabolic TNM classification. This approach may provide complementary value for clinical assessment. In addition to potential time savings for NM physicians, the tool enables pre-reporting segmentation and quantification per tumor class or whole-tumor burden and could be readily integrated into PACS workflows. Even so, prospective validation is warranted.

## Conclusion

The proposed DL-based aiding framework demonstrated the ability to map BC involvement by localizing the four key tumor regions with moderate to good accuracy. Resulting staging was highly concordant with NM physician assessment. Although expert supervision remains essential, this tool may support more straightforward and time-efficient [¹⁸F]FDG PET/CT–based staging, while enabling class-wise lesion quantification with potential prognostic value. Finally, particularly for N3 and M1 classification, high concordance was observed between clinical, NM-based and DL-based metabolic staging. These results demonstrate the tool’s high reliability in detecting advanced regional and distant disease. The approach offers a pathway toward standardization across clinical centers and provides a solid foundation for future multicenter validation studies.

## Supplementary Information

Below is the link to the electronic supplementary material.


Supplementary Material 1 (DOCX 342 KB)


## Data Availability

The dataset generated during and/or analysed during the current study is available from the corresponding author on reasonable request.
