# Supplementary material for: Automatic metabolic breast cancer staging using [¹⁸F]FDG PET/CT: comparison with nuclear medicine physician-based and clinical staging
Source: Eur J Nucl Med Mol Imaging. 2026 Jun 19;53(11):6339–50. doi: 10.1007/s00259-026-08005-y (PMC13525970; doi:10.1007/s00259-026-08005-y)
Supplement: Supplementary file 1 [file 259_2026_8005_MOESM1_ESM.docx]

**Results**

- NM-based metabolic versus clinical staging

When compared with clinical staging, metabolic NM-based staging showed concordance in 63%, 80%, and 99% of patients for T, N, and M staging, respectively. It should be noted that clinical staging already incorporates [¹⁸F]FDG PET/CT reports, which is particularly relevant for cN3 and M1 classification. There were three bilateral BC patients in the test set; therefore, T and N staging were evaluated in 103 cases. Specifically in these three bilateral cases, there were differences only in T and N in one patient (downstage from cT3 to T1 in the left primary tumor side, and upstage from cN0 to N1 in the other primary tumor side). Table S1 presents the sensitivity and specificity for stage classification of the NM-based staging relative to clinical staging. The corresponding confusion matrices for T, N and M classifications are in Figure S1.

Clinical T staging is essentially defined with MRI. Compared with clinical staging, NM-based metabolic T staging resulted in upstaging of cT1 in 12 cases and cT2 in 8 cases, corresponding to an overall T upstaging in 19% of BC cases. Conversely, T downstaging occurred in 17% of cases, including 14 cT3/cT4 cases and 4 cT2 cases.

In NM-based N staging, upstaging from cN0 to N1 occurred in 9 cases. Notably, in 4 of these 9 cases, post-surgical specimen analysis revealed the presence of positive axillary lymph nodes, although the biopsy performed at the time of staging had been negative. Only two cases were upstaged from cN1 or cN2 to N3. Overall, N upstaging was observed in 12% of cases. No BC cases were downstaged from cN3; however, cN downstaging occurred in 6 cases (5 patients were downstaged from cN2 to N1 and 1 patient from cN1 to N0). The patient downstaged from cN1 to N0 with metabolic information had undergone prior biopsy of the lymph node before the [¹⁸F]FDG PET/CT scan, and consequently, at the time of [¹⁸F]FDG PET/CT scan, only a biopsy clip remained at the site of the previously positive node.

In NM-based M staging, only one patient was upstaged to M1 when relying solely on metabolic information. This case involved a patient with high uptake in the contralateral axilla, considered metabolically suggestive of distant metastasis, but clinically attributed to post–COVID-19 vaccination.

**Table S1** Results of multiclass accuracy for T, N, and M staging, as well as sensitivity and specificity calculated per T, N, and M subclassification, derived from NM-based assessment compared with clinical staging.

|  | Sensitivity | Specificity | Multi-class accuracy  (95% CI) |
| --- | --- | --- | --- |
| T stage | | | |
| T1 | 0.45 | 0.91 | 0.63  (0.53; 0.72) |
| T2 | 0.74 | 0.62 |  |
| T3/T4 | 0.58 | 0.86 |  |
| N stage | | | |
| N0 | 0.68 | 0.99 | 0.80  (0.70; 0.87) |
| N1 | 0.91 | 0.71 |  |
| N2 | 0.33 | 0.98 |  |
| N3 | 1.00 | 0.98 |  |
| M stage | | | |
| M0 | 0.99 | 1.00 | 0.99  (0.95; 1.00) |
| M1 | 1.00 | 0.99 |  |

**Fig. S1** Confusion matrices between clinical staging (reference) and NM physician-based metabolic staging (predicted class) for T, N, and M staging in the 103 primary tumors (subfigures A, B, and C, respectively). Note that for T staging, 102 cases were used (lobular carcinoma in situ excluded; TN staging not defined); for N staging, only 101 cases were included (1 lobular carcinoma in situ without TN staging defined, and an Nx); for M staging, all 103 cases were included.
